# Kinetics and persistence of anti‐SARS‐CoV‐2 neutralisation and antibodies after BNT162b2 vaccination in a Swiss cohort

**DOI:** 10.1002/iid3.583

**Published:** 2021-12-29

**Authors:** Lara Šošić, Marta Paolucci, Agathe Duda, Fabio Hasler, Senta M. Walton, Thomas M. Kündig, Pål Johansen

**Affiliations:** ^1^ Department of Dermatology University of Zurich Zurich Switzerland; ^2^ Department of Dermatology University Hospital Zurich Zurich Switzerland; ^3^ Research & Development Saiba Biotech Pfaeffikon Switzerland

**Keywords:** antibody response, BNT162b2, COVID‐19, mRNA vaccine, neutralisation assay, SARS‐CoV‐2

## Abstract

**Introduction:**

Since the emergence of severe acute respiratory syndrome coronavirus 2 (SARS‐CoV‐2), substantial effort has been made to gain knowledge about the immunity elicited by infection or vaccination.

**Methods:**

We studied the kinetics of antibodies and virus neutralisation induced by vaccination with BNT162b2 in a Swiss cohort of SARS‐CoV‐2 naïve (*n* = 40) and convalescent (*n* = 9) persons. Blood sera were analysed in a live virus neutralisation assay and specific IgG and IgA levels were measured by enzyme‐linked immunoassay and analysed by descriptive statistics.

**Results:**

Virus neutralisation was detected in all individuals 2–4 weeks after the second vaccine. Both neutralisation and antibodies remained positive for >4 months. Neutralisation and antibodies showed positive correlation, but immunoglobulin G (IgG) and immunoglobulin A (IgA) seroconversion took place 2–4 weeks faster than neutralisation. Spike‐protein specific IgG levels rose significantly faster and were more stable over time than virus neutralisation titres or IgA responses. For naïve but not convalescent persons, a clear boosting effect was observed. Convalescent individuals showed faster, more robust and longer‐lasting immune responses after vaccination compared to noninfected persons. No threshold could be determined for spike protein‐specific IgG or IgA that would confer protection in the neutralisation assay, implicating the need for a better correlate of protection then antibody titres alone.

**Conclusions:**

This study clearly shows the complex translation of antibody data and virus neutralisation, while supporting the evidence of a single dose being sufficient for effective antibody response in convalescent individuals.

## Graphical abstract

The kinetics of antibodies and virus neutralisation induced by vaccination with BNT162b2 was studied. Neutralisation and antibodies remained positive for >4 months. Immunoglobulin G (IgG) and immunoglobulin A (IgA) seroconversion took place 2–4 weeks faster than neutralisation. IgG levels rose significantly faster and were more stable over time than virus neutralisation titres or IgA responses. Boosting effect was observed in naïve but not in convalescent persons. Convalescent individuals showed faster, more robust and longer‐lasting immune responses after vaccination compared to noninfected persons.

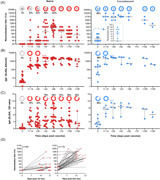

**Abbreviations**
BNT162b2: BionTech‐Pfizer mRNA vaccine against COVID‐19
COVID‐19: Coronavirus disease 2019
ELISA: Enzyme linked immunosorbent assay
PFU: plaque forming units
SARS‐COV‐2: respiratory syndrome coronavirus 2

## 1 INTRODUCTION

Vaccines against severe acute respiratory syndrome coronavirus 2 (SARS‐CoV2) are being administered worldwide with the goal to limit transmission rates and lower morbidity and mortality caused by Coronavirus disease 2019 (COVID‐19). The protection achieved with vaccination is associated with the induction of a humoral immune response with a rise in neutralizing antibody titres against the spike glycoprotein, which protrudes the virus envelope.^1^, ^2^ The antibody response has been ascribed to an early protection against infections, while T‐cell responses, next to providing immunological memory, have been associated with protection against the development of severe forms of COVID‐19 and morbidity.^3^, ^4^, ^5^ Meanwhile, public vaccination with messenger RNA  (mRNA)‐based and Adenovirus‐vectored COVID‐19 vaccines has been performed for nearly 1 year, and data on persistence of the induced immune responses are emerging. The understanding of the dynamics of antibody responses and of virus neutralisation as part of the immunological memory postvaccination can provide valuable information for the assessment of the risk of reinfection and the durability of protection. Moreover, the long‐term data will also instruct on decisions regarding further boosting, beyond the current two‐dose schedule. Therefore, to be able to predict how changes in immunity induced through either vaccination or natural infection might affect clinical outcome of SARS‐CoV‐2 infection, an immunological correlate of protection is needed. Studies investigating antibody titres after exposure to virus have shown long‐term persistence of virus‐specific antibodies for several months after infection.^6^, ^7^, ^8^ However, neutralising antibody levels seem to be more predictive of immune protection from symptomatic SARS‐CoV‐2 infection then serology alone.^9^

In this study, the kinetics of virus neutralisation and antibody responses in serum after vaccination with mRNA BNT162b2 (BioNTech/Pfizer) was investigated in a Swiss cohort. A fraction of the cohort represented convalescent persons who had recovered from COVID‐19, a situation that clearly affected antibody levels and kinetics.

## 2 METHODS

### 2.1 The study cohort

The study cohort consisted of 53 employees at the Department of Dermatology, University Hospital of Zurich. Potentially eligible persons were contacted by email and enrolled from January to April 2021 to receive two doses of the mRNA vaccine BNT162b2 (BioNTech/Pfizer) with a 28‐day interval, or only one dose for part of the convalescent individuals. The study subjects were between 21 and 61 years of age. Exclusion criteria were a known immune suppression due to medication or disease, not receiving two vaccine doses, or not providing blood samples beyond day 28 of first vaccination. All vaccinations were provided and performed by trained personnel at the University Hospital of Zurich. The study subjects were asked to provide blood samples at baseline, at the time of second vaccination session, as well as 2 and 6 weeks postsecond vaccination. In addition, they were allowed to provide in‐between and later blood samples ad libido.

### 2.2 Virus neutralisation assay

The neutralisation of SARS‐CoV‐2 was analysed in a simplified *tissue‐culture infection dose* assay, as recently described.^10^ A synthetically reconstructed and fully functional wild‐type strain of the Munich virus isolate (SARS‐CoV‐2/München‐1.1/2020/929) was used.^11^ The experiments with live virus in a biosafety level 3 lab were approved by the Swiss Federal office for the Environment (ECOGEN A202907/3). Briefly, 2 × 10^4^ VERO cells clone E6 (CRL‐1586 from CLC GmbH) were grown overnight to approximately 80%–90% adherence in flat‐bottom 96‐well cell culture plates. SARS‐CoV‐2 particles (200 PFU/well) were then mixed in round‐bottom 96‐well titre plates with twofold serial dilutions of serum and incubated at 37°C for 1 h; serum was not heat‐inactivated before the assay as we recently demonstrated that heat‐inactivation was not having an effect on virus neutralisation using the described method.^10^ The virus‐serum mixture was then added to the VERO‐E6 cell (100 PFU/ml), and the cultures were incubated at 37°C. After 3 days, the cultures were fixed by addition of paraformaldehyde and stained with crystal violet for visualization of cytotoxicity. The highest serum dilution preventing infections of the cells was defined as the neutralisation titre.

### 2.3 Enzyme‐linked immunoassay (ELISA) for detection of SARS‐CoV‐2‐specific immunoglobulin G (IgG) and immunoglobulin A (IgA)

Serum was analysed for virus specific IgG and IgA using ELISA kits from Euroimmun (Kriens) according to the manufacturer's instruction. IgG was determined with the quantitative Anti‐SARS‐CoV‐2 Quantivac kit (#EI 2606‐9601‐10G) and IgA was determined with the semi‐quantitative Anti‐SARS‐CoV‐2 kit (#EI 2606‐9601 A). The kits determine antibodies against the spike‐1 protein. The sera were not diluted and not heat‐inactivated before testing. The developed 96‐well plates were analysed by reading absorbance at 450 nm using an ELx808 ELISA reader from BioTek Instr. Inc.  The IgG results were measured as RU/ml, but expressed as BAU/ml according to WHO International Standard for COVID‐19 serological tests (1 RU/ml = 3.2 BAU/ml).^12^ The IgA results were expressed as an optical density ratio against an internal kit calibrator. Seroconversion was defined as an IgG concentration of more than 64 BAU/ml or an IgA ratio >1.1.

### 2.4 Statistics

All statistical tests were performed in GraphPad Prism (v8.0.0), except for the multivariate linear regression analysis, which were done in RStudio (v4.1.0, RStudio Team (2021)). The analysis was primarily descriptive with SARS‐CoV‐2 antibodies and neutralisation as a function of time in days after vaccination. The data was stratified for individuals having contracted a SARS‐Cov‐2‐infection (convalescent) or not (naïve) in the past year. Data was also stratified for gender and for age. The type of tests performed are indicated in the figure legends. Virus neutralisation data are illustrated as geometric means with 95% confidential intervals of the means, and non‐parametric Kruskal–Wallis tests with Dunn's test for multiple testing were applied to compare samples irrespective of the sample size. Antibody data are illustrated as medians with 95% confidential intervals and the statistical analysis was made by one‐way ANOVA with Bonferroni corrections for multiple testing. Spearman's coefficient (one‐tailed) with 95% confidence intervals and P values were calculated to evaluate the correlation between virus neutralisation and the level of anti‐spike IgG or IgA. A linear mixed model with random intercept was run with “log2(neutralisation)”, “IgG”, and “IgA” as dependent variables. Time postvaccination, SARS‐Cov‐2‐infection status (naïve or convalescent) and their interaction were fixed effects. Cubic B‐splines were added to relax assumption of linearity. The package “nlme”^13^ was used to compute the linear mixed model. The package “ggeffects”^14^ was used to compute the predicted values. All *p* values lower than .05 were considered statistically significant and *p* values >.0001 were indicated as exact numbers.

## 3 RESULTS

### 3.1 Study population

The study enrolled 53 participants, of which 33 (62%) were female and 20 (38%) were male. The baseline characteristics are summarized in Table 1. The mean age was 37 ± 11 years (21–61 years) years. Nine (17%) of the individuals were convalescent of COVID‐19 before vaccination, as confirmed by positive PCR and by sero‐positivity in antibody ELISA.

**Table 1:** Baseline characteristics of 53 study subjects vaccinated with BNT162b2

Characteristic	Naïve subjects	Convalescent subjects
Study subjects, intention to treat	44	9
per protocol	39	9
Age, median years (range)	35 (21–61)	40 (29–60)
of which <50 years	33	6
of which 50 years or older	6	3
Sex, *n* (%)		
male	15 (34%)	5 (56%)
female	29 (66%)	4 (44%)
Vaccine doses		
one	1	4
two	43	5
Median time interval, days (range)	28 (22–31)	28 (28–29)
Follow‐up time, days (range)	77 (27–149)	59 (24–131)
Postvaccination blood samples, numbers (range)	4 (1–8)	2 (1–4)

Out of the 44 naïve subjects enrolled, three subjects were excluded because they did not provide blood samples beyond day 28 of vaccination, and one subject who received one vaccination only was excluded too. The median age of the included per‐protocol study subjects was 35 years (21–61 years) of which 14 (35%) were men and 26 (65%) women. The study subjects received two vaccine doses with a median time interval of 28 days (22–31 days) and were followed up for a median of 77 days (27–149 days).

The nine COVID‐19 convalescent study subjects had a median age of 40 years (29–60 years) of which 5 (56%) were men and 4 (44%) women. Four subjects received one vaccine, while five subjects also received a booster dose with a time interval of 28–29 days. The study subjects were followed up for a median of 59 days (24–131 days). Overall, the study subjects provided on average three^1^, ^2^, ^3^, ^4^, ^5^, ^6^, ^7^, ^8^ postvaccination blood samples in addition to a baseline sample.

All vaccinated individuals developed humoral immune responses and neutralisation titres against SARS‐CoV‐2

**Figure 1:**
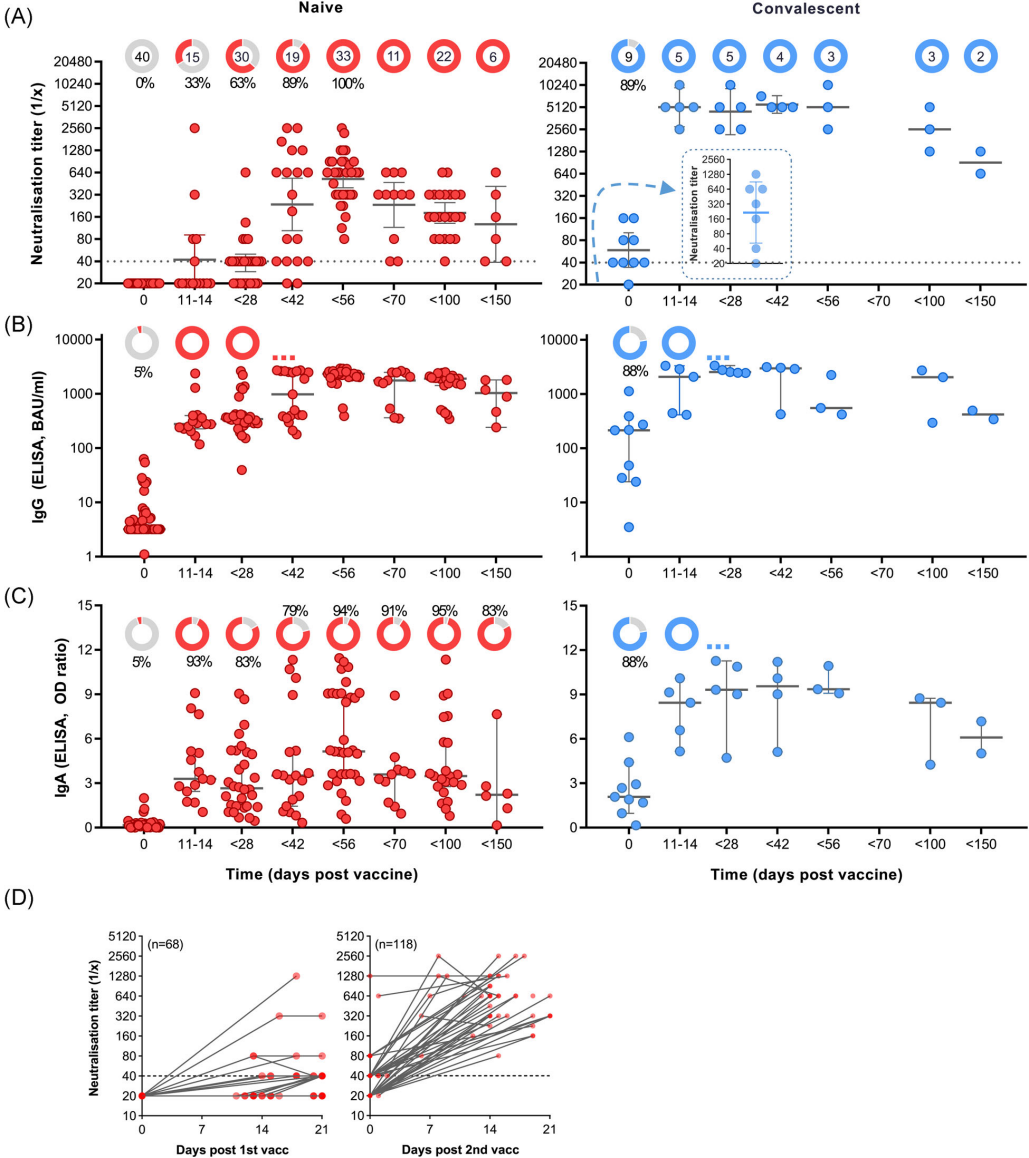
Fast and strong SARS‐CoV‐2 neutralisation after vaccination with BNT162b2 in convalescent persons. Two cohorts of naïve (left panel/red; *n* = 40) and convalescent (right/blue; *n* = 9) were defined based on history of COVID‐19 before first vaccination (Day 0). Secondary vaccination was done approximately 4 weeks after the first vaccine dose. Convalescent subjects received first vaccination 3–12 months after infection. (A) Reciprocal titres of SARS‐CoV‐2 neutralising antibodies measured using a tissue‐culture infection dose‐based method. The donut charts illustrate the fraction of persons with positive (red or blue) virus neutralisation at time points indicated in the figures underneath. The numbers in the donut centres indicate the number of persons analysed at the given time point. The inset (right panel) represents neutralisation titters of convalescent individuals 4–12 weeks postinfection, but before vaccination. Neutralisation titres equal or bigger than 40, as indicated by the dashed line, are considered as positive. (B) Spike (S1) protein‐specific IgG in BAU/ml as measured by ELISA. (C) Spike protein‐specific IgA in in OD‐ratio and as measured by ELISA. (D) Virus neutralisation titres of serum from naïve participants was measured as a function of time after the first (left panel) and the second (right panel) vaccination. Neutralisation data are illustrated as geometric means with 95% confidential intervals of the means, while IgG and IgA data are illustrated as medians with 95% CI. Nonparametric Kruskal–Wallis test with Dunn's test were applied to compare samples at each time point with the following one. All *p* values lower than 0.05 were considered statistically significant and *p* values >0.0001 were indicated as exact numbers. COVID‐19, coronavirus disease 2019; ELISA, enzyme‐linked immunoassay; IgA, immunoglobulin A; IgG, immunoglobulin G; SARS‐CoV‐2, severe acute respiratory syndrome coronavirus 2 [Color figure can be viewed at wileyonlinelibrary.com]

Vaccinated blood donors involved in the study (*n* = 49) were divided into two different cohorts according to their immune status before vaccination. Their virus neutralisation titres and the development of anti‐SARS‐CoV‐2 IgG and IgA antibodies as a function of time were determined (Figure 1). The first cohort comprised 40 subjects that were naïve to SARS‐CoV‐2 virus (red), and the second compromised 9 convalescent individuals with a COVID‐19 history 3–12 months before vaccination (blue). Study participants donated blood before immunisation with mRNA vaccine BNT162b2 (Day 0) and at different time intervals after vaccination (days 11–150).

Figure 1A illustrates the virus neutralisation in the two cohorts, and the donut charts in the upper part of the graphs indicate the total number of individuals that donated blood at this time point and the percentage of individuals with a positive test reaction; neutralisation titres equal or greater than 40 were considered as positive. In the naïve group no virus neutralisation could be determined at baseline (Figure 1A, left panel). Within 14 days of the primary vaccination, SARS‐CoV‐2 neutralisation could be detected in 33% of the tested individuals, while 63% of the tested individuals had developed neutralisation sera before day 28. The median reciprocal neutralisation titre by the time of the second vaccination was 40, with 5 out of 30 (17%) having titres of 80 to 640. Effective SARS‐CoV‐2 neutralisation was determined in 100% of the participants 2–4 weeks after the second injection (Days 42–56), and the median reciprocal titre had increased to 640 (80–2560). Indeed, while the primary vaccination produced a delayed and a relatively weak neutralisation effect within 3 weeks, the data revealed a clear boosting effect of the BNT162b2 vaccine (Figure 1D). After Day 56, a decline in virus neutralisation of sera was observed, but all tested sera remained positive within 150 days of testing (Figure 1A). By Day 100, the median neutralisation titre was 160 (*n* = 22), and by Day 150, the median titre was 113 (*n* = 6).

### 3.2 Vaccination of convalescent patients results in very high and long‐lasting neutralisation antibodies

Convalescence from COVID‐19, as for other infections, is typically associated with a polyclonal antibody response, including protection from reinfection through pathogen neutralisation. Out of the nine convalescent individuals included in the current study, postinfection but prevaccination sera were available from seven persons. In one out of seven, no virus neutralisation could be detected 4–12 weeks postinfection, while for six out of seven, maximum reciprocal neutralisation titres of 40–1280 (median 320) were measured (Figure 1A, right panel, inset). By the time of vaccination, 8 out of 9 (89%) of the convalescent study subjects showed positive virus neutralisation, but the median neutralisation titre was reduced to 20–160 with a median of 40 (Figure 1A, right panel). However, the vaccination resulted in a very fast increase in virus neutralisation in sera from all study subjects, with median reciprocal titres reaching 5120 (2560–10240) by Days 11–14. The virus neutralisation capacity of the serum samples remained stable for at least 6 weeks, where after a decline was observed. In the time period of 70–150 days after vaccination, neutralisation titre remained above 640 for all serum samples. No boost of neutralisation was observed in the convalescent study subjects that received a second vaccination after 4 weeks.

### 3.3 Complete anti‐spike IgG seroconversion precedes virus neutralisation with 6 weeks

None of the naïve study subjects, but 8 out of 9 (89%) of the convalescent subjects presented with SARS‐CoV‐2‐specific IgG above the threshold before vaccination (Figure 1B). Within 2 weeks of a single vaccine dose, anti‐spike IgG seroconversion was observed in all vaccinated individuals (*p* < .0002), regardless of the immune status at baseline. The IgG concentrations were only slightly higher in sera from convalescent subjects than in sera from naïve subjects. Secondary vaccination in the naïve cohort caused a further rise in anti‐spike IgG (*p* < .0001), with a peak by Day 56, but the booster effect was less evident than for the boosting of virus neutralising antibodies (Figure 1A vs. 1B, left panel). Finally, the spike‐specific IgG levels for the cohort showed stable levels of over time, with no significant decay after day 56, although it should be noted that only 11, 22, 6 sera were available for the time periods 56–69, 70–99, and 100–150 days, respectively.

Also, spike‐specific IgA seroconversion was reached much faster than the neutralisation titres in the naïve cohort, with 93% being positive by Days 11–14 (Figure 1C, left panel). Although all study subjects developed seroconversion at some point after vaccination (details not shown), the cohort was at no time point completely seroconverted. Indeed, spike‐specific IgA in the naïve individuals showed a much greater interindividual variability as compared to the IgG levels as well as neutralisation titres. In contrast, all individuals that had recovered from COVID‐19 had detectable spike‐specific IgA levels at all time‐points assessed after vaccination, and as for IgG and neutralisation, the effect peak was reached within 2 weeks of primary vaccination (Figure 1C, right panel).

### 3.4 COVID‐19 convalescent individuals showed a stable neutralisation capacity over time although the stability of anti‐SARS‐CoV‐2 antibodies was comparable between convalescent and naïve group

**Figure 2:**
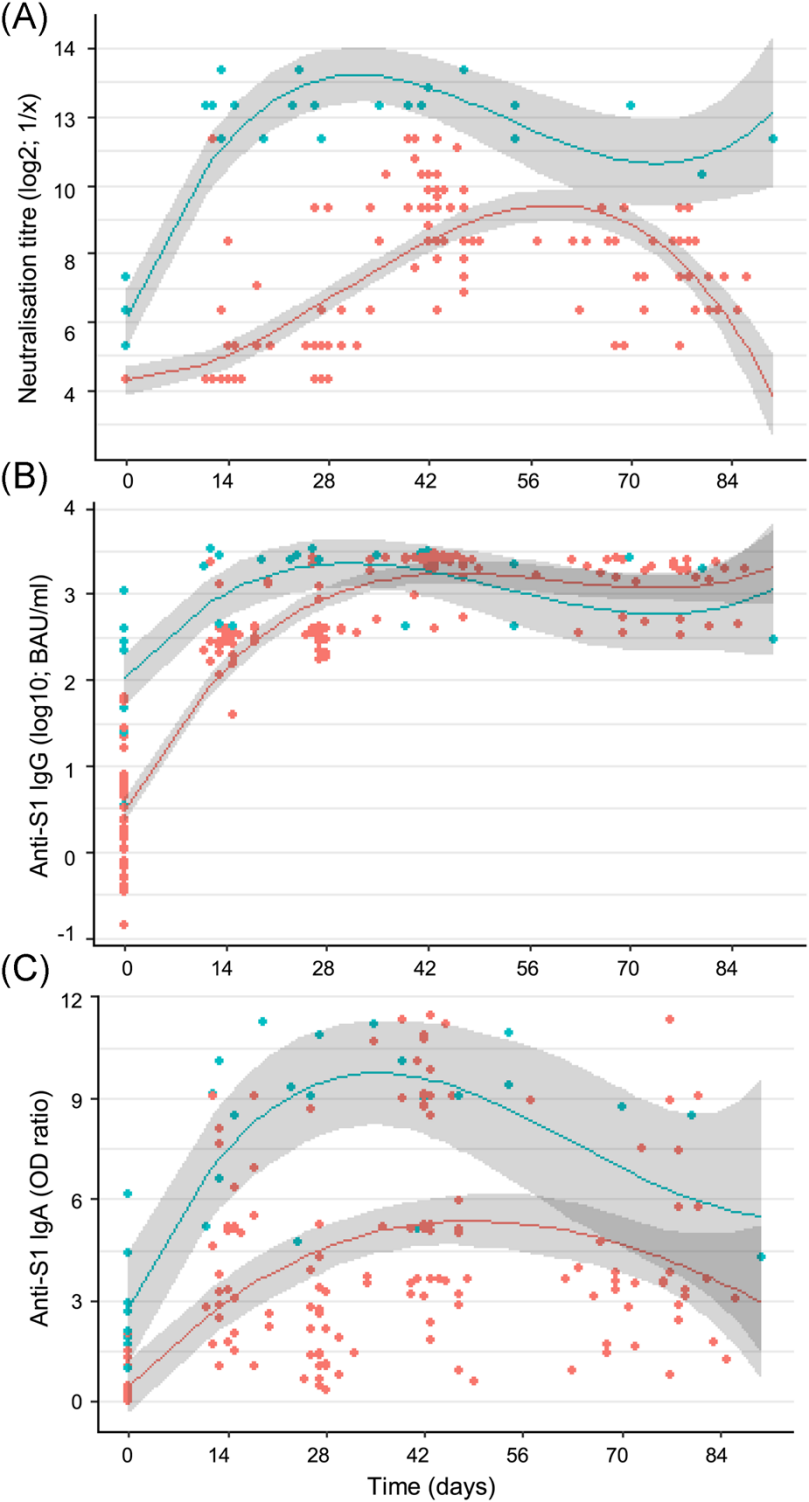
Modelling of virus neutralisation and antibodies kinetics. Anti‐SARS‐CoV‐2 neutralisation (A), IgG response (B), and IgA response (C) are stratified in convalescent (blue) and naïve (red) according to the COVID‐19 history of the participants before vaccination on Day 0 and approximately 4 weeks later. Serum samples collected within 100 days of the primary vaccination were included in the analysis. Regression curves (lines) and 95% CI (shades) are shown. CI, confidence interval; COVID‐19, coronavirus disease 2019; IgA, immunoglobulin A; IgG, immunoglobulin G; SARS‐CoV‐2, severe acute respiratory syndrome coronavirus 2 [Color figure can be viewed at wileyonlinelibrary.com]

A linear mixed‐model regression analysis was done to model the kinetics of SARS‐CoV‐2 neutralisation and spike‐specific antibodies for the naïve and convalescent cohorts (Figure 2); only data until day 100 postvaccination was included, as only a limited number of samples for the later time points was collected. In the naïve group, peak neutralisation capacity was reached after the second vaccination at approximately days 40–50, after which a time‐dependent decline in the neutralisation was observed. In the convalescent cohort, peak neutralisation titres were reached by approximately 20–25 days, with no notable effect of a second vaccination. The neutralisation titres in the convalescent cohort remained high through approximately Day 70, while the modelled titres in the naïve cohort showed an earlier decay, although peaking later. For the modelled IgG responses, the increase after first vaccination was comparable in the two cohorts, and the antibody levels were comparably high and stable across approximately 80–85 days. The modelled spike‐specific IgA antibodies in serum from vaccinated naïve and convalescent study subjects followed an intermediate pattern as compared to neutralisation capacity and IgG levels. IgA peaked 1–2 weeks earlier in the convalescent than in the naïve cohort. In the naïve cohort, the IgA levels around day 85 were approximately 55% of the peak levels.

### 3.5 Positive correlation between the virus neutralisation capacity and the humoral responses

**Figure 3:**
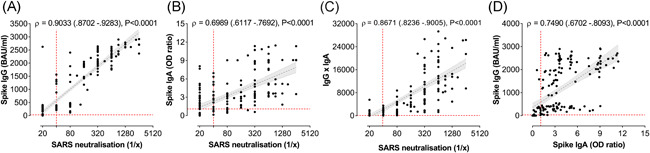
Strong correlation between SARS‐CoV‐2‐specific IgG and IgA antibodies. Correlation analyses are performed in the naïve cohort and spike protein‐specific IgG and IgA antibody concentration and anti‐SARS‐CoV‐2 neutralisation titres are compared for each person at each blood donation (*n* = 176). Correlation plot between reciprocal neutralising titres and spike‐protein‐specific IgG (A) or IgA (B) and antibody titres. (C) Correlation plot between neutralisation and a combined IgG and IgA factor (the product of IgG and IgA concentrations). (D) Correlation plot of IgG and IgA. Spearman correlation coefficients (ρ), 95% CI values and P values are indicated above the graphs and plotted as hatched linear regression lines with shaded 95% CI. CI, confidence interval;IgA, immunoglobulin A; IgG, immunoglobulin G; SARS‐CoV‐2, severe acute respiratory syndrome coronavirus 2 [Color figure can be viewed at wileyonlinelibrary.com]

SARS‐CoV‐2‐specific IgG and IgA levels in serum of naïve study subjects were compared with the ability of donor serum to neutralize the virus in vitro. A very strong positive correlation (ρ = 0.9033, *p* < .0001) between IgG levels and neutralisation was observed (Figure 3A). Especially for neutralisation titres >160, the correlation between IgG and neutralisation was evident, whereas for neutralisation titres of 40 and 80, the IgG levels were highly variable and correlated less with the degree of virus neutralisation. Indeed, at neutralisation titres below detection level (<40), sera with a wide range of positivity for spike protein‐specific IgG could be found. Also, spike‐specific IgA showed a significant positive correlation with SARS‐CoV‐2 neutralisation (Figure 3B), although less strong as compared to the IgG (ρ = 0.6989). A strong correlation was also observed between neutralisation and a combined IgG and IgA score (Figure 3C; ρ = 0.8671) as well as between IgG and IgA (Figure 3D; ρ = 0.7490). The latter test suggested that there were two populations of IgA responders, which could be distinguished based on their IgG response (high or low).

### 3.6 The neutralising capacity of anti‐SARS‐CoV‐2 antibodies was independent of age and gender of the vaccinated individuals

**Figure 4:**
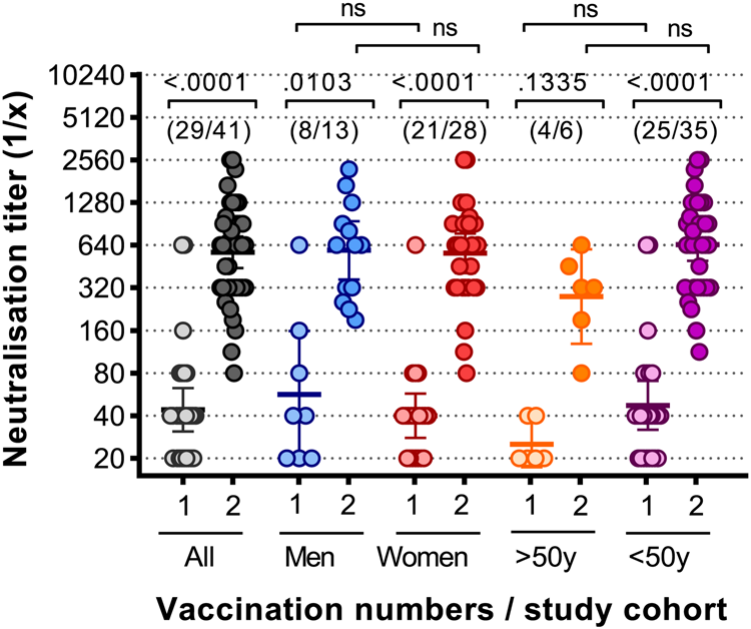
Peak virus neutralisation titres of naïve individuals after first and after second vaccination are independent of gender and age. Virus neutralisation titres are shown after the first^1^ and the second^2^ vaccination for all naïve individuals and represented also according to gender (men/women) and age (>50 years or <50 years). The numbers in bracket in the upper part of the graph indicate in each group the number of persons that donated blood after the first and after the second dose. Statistical analysis was performed using Kruskal–Wallis test (nonparametric one‐way ANOVA) with Dunn's multiple comparison test. All *p* values lower than .05 were considered statistically significant and *p* values >.0001 were indicated as exact numbers. ANOVA, analysis of variance [Color figure can be viewed at wileyonlinelibrary.com]

Naïve study participants were also divided into different sub‐groups according to gender (male/female) and age (younger or older than 50 years). Serum neutralisation titres were measured and compared after the first and after the second vaccination dose in all study participants as well as within the different study sub‐groups. A significant increase in SARS‐CoV‐2 neutralisation titres between the first and second BNT162b2 vaccine injection was observed in the entire naïve study cohort (*p* < .0001 by Kruskal–Wallis and Dunn's tests), in men (*p* = .103), women (*p *< .0001) and study participants below 50 years of age (*p* < .0001). For study participants above 50 years of age, a clear increase in boosting effect was observed after second vaccination, but the effect did not reach statistical significance. Of note, only 6 study subjects older than 50 years of age were included in the study impeding drawing definite conclusion on the impact of age on the vaccine induced immunity. When the neutralisation potential of anti‐SARS‐CoV‐2 antibodies after the first and the second vaccination dose was compared between the sub‐groups, no significant differences were observed although a clear trend was that observed neutralisation titres were higher in the younger compared to the older age group after first and second vaccination (Figure 4).

## 4 DISCUSSION

This study aimed at investigating the kinetics of both SARS‐CoV‐2 binding and neutralising antibody responses in COVID‐19‐vaccinated employees at a Swiss University. The results show that all per‐protocol participants developed a clear positive neutralisation response to SARS‐CoV‐2, with a parallel rise in anti‐S1‐IgG and anti‐S1‐IgA antibodies, confirming previously published data on antibody kinetics after COVID‐19 vaccination.^1^, ^2^

The naïve study subjects showed no virus neutralisation at base line, whereas eight out of the nine convalescent study subjects had neutralising antibodies before administration of the first vaccination dose. Notably, in convalescent individuals, the titre of SARS‐CoV‐2 neutralising antibodies increased significantly faster than in naïve individuals, with peak neutralisation being reached 1–2 weeks after first vaccination in convalescent and 2–4 weeks after second vaccination in naïve individuals, hence, a time difference of approximate 4−7 weeks. In convalescent subjects, these findings are thought to be attributed to long‐lived plasma cells and class‐switched memory B cells, re‐activated by vaccination to rapidly mount virus‐specific antibody responses.^15^ In naïve subjects, the B cells need to be primed first, undergo affinity maturation, and differentiate into plasma cells and memory cells, which then are reactivated upon second vaccination.^15^ This prime‐boost mechanism for stimulation of antibody responses is the same as described for other vaccines, e.g., childhood vaccines.

Interestingly, while primary vaccination in naïve subjects had no detectable effect on virus neutralisation within 2 weeks of injection (reciprocal median titre <40) and only little further effect after 4 weeks (median = 40), a strong IgG response was determined in all individuals within 2 weeks of the priming dose. This phenomenon can be explained by two different mechanisms. First, it may suggest that BNT162b2 stimulates a broad spectrum of spike specific IgG antibodies of which not all are having neutralising capacity. Indeed, antibodies may have various functions,^16^ and although neutralisation is a correlate of protection for many viral diseases,^17^ not all antibodies that bind a virus will neutralise it. For a given antigen, antibodies may neutralise (e.g., influenza hemagglutinin), bind the antigen on the virus surface and thereby perhaps slow the spread of infection without being neutralising (e.g., influenza neuraminidase),^18^ or in some instances even enhance the infection (e.g., dengue virus).^19^ Noval et al.^20^ recently showed that the antibody isotype diversity against SARS‑CoV‑2 was associated with differential serum neutralisation capacities in 101 convalescent patients.^20^ Most of the recovered persons generated antibodies with low neutralisation capacity, with only 6% showing high virus neutralising titres. Higher combined IgG, IgM, and IgA levels correlated well with neutralisation, while individuals with positive IgG alone showed poor neutralisation response. These results suggested that a broader repertoire of antibodies may contribute to better virus neutralisation. The current study could not confirm these results, since the correlation of neutralisation and a combined IgG and IgA score did not improve as compared to the correlation of neutralisation and IgG alone. A second possible reason for the high IgG concentration without virus neutralisation 2 weeks post primary vaccination may also be the lack of affinity maturation of the first wave of secreted antibodies. This phenomenon is known from childhood vaccines, but also in COVID‐19 convalescent individuals, where virus neutralising antibodies were shown to change as a result of accumulated somatic mutations over months.^21^, ^22^ Muecksch et al.^23^ also showed that increasing antibody diversity through prolonged or repeated antigen exposure improved protection against diversifying SARS‐CoV‐2 populations.^23^ This effect may have consequences for the protection against SARS‐CoV‐2 variants, as antigenic drifts in virus variants may facilitate escape from neutralising antibodies. However, antibody maturation may also potentiate cross‐neutralising ability to circulating variants, suggesting that declining antibody levels may not be indicative of declining protection.^24^ Indeed, maturation of the IgG antibodies after infection and booster vaccination have been demonstrated by measuring the anti‐SARS‐CoV‐2 IgG avidity.^25^, ^26^

In addition to an earlier onset after vaccination, we found that peak neutralisation titres in convalescent study subjects were much higher than in naïve subjects. Additionally, after approximately 60 days, the neutralising antibodies of naïve individuals decreased, whereas for convalescent patients they remained high, suggesting, therefore, a more robust and long‐lasting protection in the latter cohort, as it has been implicated previously.^27^ Five out of nine convalescent study subjects received a second dose of BNT162b2, but no boosting of the neutralisation or antibody responses was observed. This result supports the assumption and general recommendation that a single dose is sufficient for effective antibody response in previously infected individuals. This is consistent with published data of antibody responses to vaccination in convalescent individuals.^28^, ^29^, ^30^ While these other studies demonstrated these effects for the dynamics of antigen‐specific antibodies, the current study demonstrated this also for the neutralising effect of the antibodies. Moreover, it has also been shown that a single mRNA vaccine dose in convalescent individuals facilitates cell‐mediated responses, including those against other variants of SARS–CoV‐2.^29^, ^30^, ^31^, ^32^, ^33^ Together with these other studies, our results clearly document that there are key differences in the vaccine immune responses and efficacy in SARS‐CoV‐2 naïve versus SARS‐CoV‐2 convalescent individuals.

While SARS‐CoV‐2 neutralisation titres reached much higher peak levels in convalescent than in naïve individuals, the IgG and IgA peak levels were more comparable in the two cohorts. Of note, we observed a higher variance of anti‐S1 IgA levels compared to anti‐S1. Moreover, IgG levels were quite stable over the time period of investigation, whereas the IgA levels were clearly decreasing after the peak at 42–56 days postvaccination, which has also been observed by others.^34^, ^35^, ^36^ Interestingly, our analysis showed that spike‐specific IgG antibodies were rather stable over time, while the neutralisation ability of these antibodies contracted much faster in the same time span. In the past, the decay in neutralising antibodies after infection or vaccination has been described in detail and support our current findings.^21^, ^23^, ^37^, ^38^, ^39^ In a recent study by Haveri et al.,^40^ long persistence of neutralising antibodies was observed after SARS‐CoV‐2 infection. Here, neutralising antibodies was determined in 89% of the patients after 13 months, with 97% positive for spike IgG, but only 36% for nucleocapsid.

A limitation of this study is the small cohort size of 49 patients (only 9 of them being convalescent), with variations in number and time point of blood collections. Concerning neutralising antibodies against emerging viral variants no statement could be made, as the neutralisation assay was carried out with only the original viral variant. Furthermore, only the humoral response was measured, with no information on cellular immune mechanisms such as antiviral T and B cell memory leading to immune protection.

In conclusion, we could show an induction of SARS‐CoV‐2 neutralising antibodies in all vaccinated individuals within 6–7 weeks after first vaccination, while spike protein‐specific antibodies were strongly induced already after 2 weeks. Peak neutralisation titres for previously infected individuals were reached within 2 weeks of the first vaccine dosing and for naïve participants within 2 weeks after the second vaccine dose, with neutralisation titres being much higher in convalescent individuals. A booster dose in the latter did not further improve neutralisation or immune responses. Consequently, this study clearly shows the complex translation of antibody data and virus neutralisation, while supporting the evidence of a single dose being sufficient for effective antibody response in convalescent individuals.

## CONFLICT OF INTERESTS

Senta M. Walton is employee of Saiba Biotech. Thomas M. Kündig is a medical advisor to Saiba Biotech, Pål Johansen has received financial support from PCI Biotech and Allergy Therapeutics. The other authors have no conflict of interests.

## AUTHOR CONTRIBUTIONS

*Concept*: Lara Šošić, Agathe Duda, Fabio Hasler, Thomas M. Kündig, and Pål Johansen. *Methodology*: Agathe Duda, Fabio Hasler, and Pål Johansen. *Experimental work*: Agathe Duda and Fabio Hasler. *Resources*, Thomas M. Kündig and Pål Johansen. *Manuscript writing and editing*: Lara Šošić., Marta Paolucci, Senta M. Walton, and Pål Johansen. *Review*: all authors. *Figure preparation*: Marta Paolucci and Pål Johansen. *Supervision*: Senta M. Walton and Pål Johansen. *Project administration*: Pål Johansen. *Funding acquisition*, Thomas M. Kündig.

## ETHICS APPROVAL AND PATIENT CONSENT STATEMENT

The study was approved by the independent ethics committee of the Kanton of Zurich (BASEC‐number: 2021‐01361) and the Data Governance Board of the University Hospital of Zurich (DUP‐251), respectively, and was conducted in compliance with the Declaration of Helsinki guidelines. The study was registered at www.clinicaltrials.gov (NCT04979871). All study subjects provided a written informed consent to blood collection and to the use, analysis, and publication of the generated data.

## Data Availability

The data that support the findings of this study are available from the corresponding author upon request. The complete data are not publicly available due to privacy or ethical restrictions.
